# Classification of colon polyps with malignant potential using statistical analysis of features extracted from *ex vivo* optical coherence tomography images

**DOI:** 10.1186/s12938-025-01443-z

**Published:** 2025-09-26

**Authors:** Christos Photiou, Andrew D. Thrapp, Guillermo J. Tearney, Costas Pitris

**Affiliations:** 1 Department of Electrical and Computer Engineering, KIOS Research and Innovation Center of Excellence, University of Cyprus, Nicosia, Cyprus; 2 Harvard Medical School, Wellman Center for Photomedicine, Massachusetts General Hospital, Boston, MA 02114 USA

**Keywords:** Optical coherence tomography, Colorectal cancer, Polyps, Feature extraction, Biomarkers, Statistical analysis

## Abstract

To reduce the burden of screening of colorectal polyps, *leave-in-situ* management of diminutive (≤ 5 mm) polyps is being considered. However, such an approach requires increased diagnostic efficacy (PIVI-1 criterion). The aim of this study was to use Optical Coherence Tomography (OCT) for the classification of pre-malignant polyps as benign (i.e., normal or hyperplastic) vs. those with a malignant potential (i.e., adenomas and sessile serrated adenomas) at an accuracy that would enable clinical screening of colorectal cancer. The OCT raw data, from volume imaging of resected polyps, were used to extract features that can serve as biomarkers of disease. In addition to intensity and textural measures, novel biomarkers, such as scatterer size, group velocity dispersion, and spectral information, were also estimated. Their statistical properties were combined to produce scores which, in turn, were used to classify the images or polyps. This approach yielded 79.6% accuracy (72.3% NPV) for the classification of individual images and 97.3% accuracy (95.5% NPV) when combining the feature values to classify whole polyp sections. The results of this study confirm the potential of OCT imaging of colorectal polyps as a viable adjunct to colonoscopy that could enable leave-in-situ management strategies.

**Supplementary Information:**

The online version contains supplementary material available at 10.1186/s12938-025-01443-z.

## Introduction

Colorectal cancer (CRC) is a leading cause of cancer deaths, with more than 356,000 new cases in 2022 in Europe [1]. In USA, approximately 150,000 new cases and 53,000 deaths are expected each year [2]. CRCs mainly develop from polyps or adenomas with dysplasia. Since polyps gradually evolve to malignancies over time, population-based screening, for examination of suspicious lesions, is both a viable and an important cancer prevention strategy. It is estimated that screening has the potential to reduce the burden of CRC, with a reduction in mortality ranging from 18 to 57% [3]. Sedated colonoscopy accounts for most of CRC screening procedures, contributing to a 35% decline in overall CRC mortality over the past two decades [4]. Since 2003, the European Council proposed the implementation of CRC screening for men and women aged 50–74 years [5]. Although colonoscopy is an effective screening technique, there is room for improvement, both in terms of cost and diagnostic effectiveness. First, the rate of incidence of CRC has increased by 8% over the last decade, increasing the number of colonoscopies required, imposing a significant financial burden to the healthcare system [2]. In addition, colonoscopy misses 20–30% of adenomas due to (i) the forward viewing endoscope that misses polyps behind the colon’s folds [6, 7] and flat serrated lesions [8], (ii) inadequate time and care spent inspecting the mucosa [6, 8], and (iii) lack of real-time feedback to the clinician, since resected polyps require histopathologic processing ex vivo. Missed lesions lead to cancers that arise in between colonoscopic screenings, accounting for about 10% of new cases [9]. Recent efforts to improve colonoscopic efficiency aim at reducing the procedure time and pathology burden by leaving diminutive polyps (< 5 mm) in place. If successful, such a leave-in-situ strategy could also result in significant cost savings [10]. However, this approach introduces additional risks, since it proposes leaving in place 80% of the polyps that are now resected. Hence, along with increased vigilance, advancements in technology are required to improve the diagnostic efficacy of colonoscopy to ensure that the risk from the polyps that are not resected is minimized. For a technology to guide colonoscopic decisions, regarding polyps ≤ 5 mm, the negative predictive value (NPV) should be equal to or exceed 90% (PIVI-1) [11].

Novel optical imaging approaches have been proposed to improve the efficacy of colonoscopy. For example, narrowband imaging (NBI), in combination with deep learning, aims to automatically detect colorectal cancer and assist the clinicians with the diagnosis. However, there are various challenges in the application and clinical acceptance of NBI, where polyps do not always clearly standout from the background mucosa [12, 13]. Detecting and classifying polyps can be addressed with Optical Coherence Tomography (OCT). OCT can rapidly and non-invasively generate three-dimensional (3D) images of tissue microstructure at a resolution of 10–20 μm. This technology has been explored as a means for optical biopsy since early in its development. However, OCT imaging in the colon has not advanced as much as other clinical applications, since, until recently, there were no endoscopic OCT systems to comprehensively image the large diameter and corrugated anatomy of this organ [14]. Even with recent system developments [15, 16], the diagnostic accuracy of OCT for colon polyp diagnosis is unknown. One of the reasons is that, thus far, the diagnosis is based on the OCT images of tissue microstructure and heuristic “radiological-like” criteria, without the functional or biochemical information required to identify early signs of cancer [17]. Some attempts to extract additional information from OCT data, e.g., inverse spectroscopic OCT and scattering coefficient maps, were not further developed [18–20]. A key limitation of inverse spectroscopic OCT is the inaccuracies in the application of the first-order Born approximation of scattering due to discontinuities at the boundaries which exist in tissue [19]. The use of the scattering coefficient is also hindered by the significant overlap between normal and cancer values due to heterogeneity within the various tissue types, variable amounts of ischemic time, and/or the health status of the patient [20]. Most deep learning studies so far have included a very small number of patients, i.e., 18–43 patients [21–23], which limits the accuracy of the techniques, despite the large number of images collected, since, unfortunately, most of the images from the same patient are highly correlated. Luo et al. utilize a vision transformer (ViT), which provides very promising results, even when applied to only 43 patients, a number that is small for the traditionally data hungry ViTs [22]. On the other hand, feature-based learning can more effectively classify such smaller datasets. Furthermore, the majority of the studies to date concentrate on the differentiation of normal tissue from invasive carcinoma [21–23]. This level of efficacy is not adequate to enable *leave-in-situ* applications. Instead, imaging must be able to delineate early pre-malignant lesions, including dysplasia.

In this study, a novel approach has been evaluated that can address some of the current limitations in the interpretation of OCT images and enable the clinical application of OCT in the screening of CRC. The aim was to enable the classification of precancerous colorectal polyps as benign (i.e., normal or hyperplastic) vs. those that exhibit a malignant potential [i.e., adenomas and sessile serrated adenomas (SSAs)]. The OCT raw data were exploited to extract features that currently remain unseen and unused but can be useful as biomarkers of disease, since they reflect both structural but also sub-cellular and biochemical changes in the tissue. In addition to intensity and textural measures, novel biomarkers, such as scatterer size, group velocity dispersion (GVD), and spectral information, were also included [24–28]. The statistical properties of these features were combined to produce scores which, in turn, were used to classify the images or sections of polyp as benign vs. malignant potential. This approach yielded 79.6% accuracy (72.3% NPV) for the classification of individual images and 97.3% accuracy (95.5% NPV) when combining the feature values to classify whole polyp sections.

## Results

**Fig. 1.**
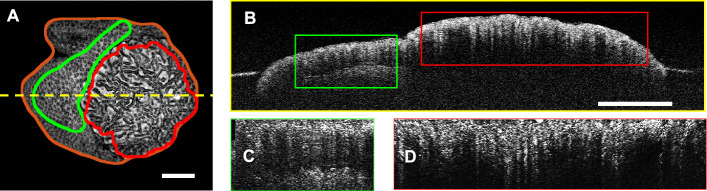
(**A**) An en face projection of a polyp created from a volume of 1000 OCT images (each 1000 A-Scans x 1024 pixels). The brown line marks the extend of the polyp. The green and red lines mark normal and adenomatous sections of the polyp as marked by an expert pathologist. (**B**) The B-Scan OCT Image corresponding to the yellow dashed line in A. The green and red rectangles correspond to the regions marked as normal and adenomatous, respectively. (**C**) The pre-processed normal section of the image. (**D**) The pre-processed adenomatous section of the image. The scale bars are 500 μm

Table S1 in the supplementary material shows the ten most significant features and their MANOVA coefficients used to create each score of a specific polyp. Figure 1 shows the distributions of the scores calculated from the combination of each type of features. In all cases, there was a statistically significant difference between the categories, based on the p value of the Student *t* test.

**Fig. 2:**
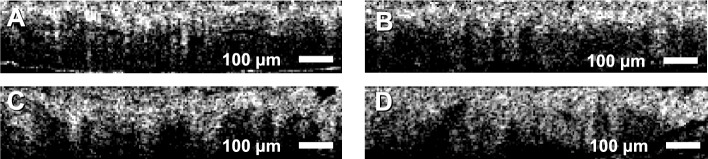
Examples Optical Coherence Tomography (OCT) sections of (**A**) normal, (**B**) hyperplastic, (**C**) adenomatous, and (**D**) sessile serrated adenomatous (SSA) polyps

**Fig. 3:**
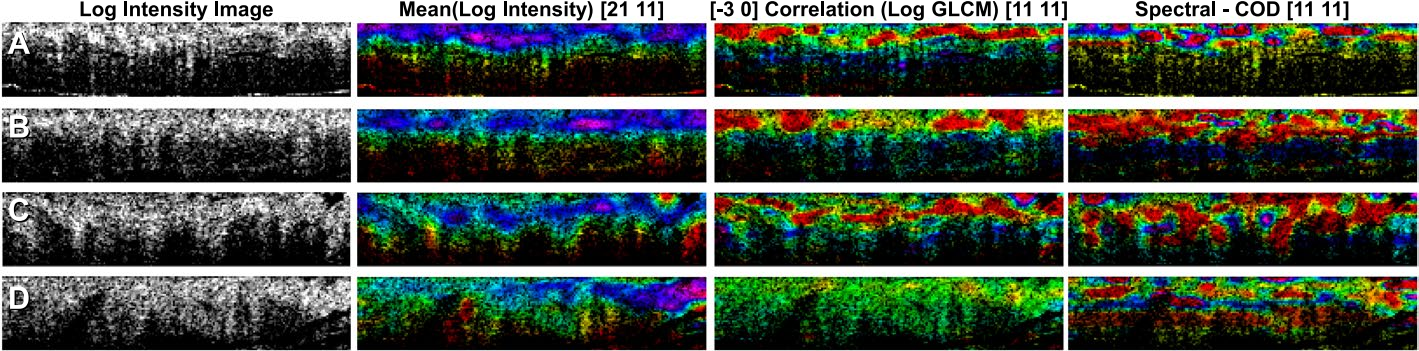
Standard logarithmic intensity images of (**A**) normal, (**B**) hyperplastic, (**C**) adenoma, and (**D**) SSA. Each column corresponds to example features calculated from non-overlapping neighborhoods of the OCT data, i.e. the mean of the intensity of the logarithmic image pixels, the correlation as calculated from the GLCM matrix at a distance of 3 pixels in the direction of increasing depth, and the bandwidth of the correlation of the derivative (COD) of the spectrum. The normalized values of each feature are represented by the color (H) over the intensity (V) of the original OCT image, in HSV format.

Figure 2 shows the distribution of the combined score for the classification of benign vs. malignant potential polyps. Based on the threshold selected, each image could be classified with an accuracy of 79.7%. The negative predictive value (NPV) was 72.3%. When the results were combined by polyp section, as described above, the accuracy was 97.3% and the NPV was 95.5% (Fig. 3). All relevant metrics of the classification are shown in Table 1.

**Table 1:** Classification performance

Metric	Classification per image	Classification per section
Accuracy	79.69%	97.25%
Area under the curve (AUC)	0.7641	0.9748
Sensitivity	86.81%	96.33%
Specificity	66.01%	98.63%
NPV	72.25%	94.74%
PPV	83.07%	99.06%

## Discussion

The results of this study confirm the potential of OCT imaging of colorectal polyps as a viable adjunct to standard colonoscopy that could enable leave-in-situ management strategies. Using a broad range of thoroughly selected features, which can serve as biomarkers of disease, and their combined scores, the polyp images were classified into benign vs. malignant potential with an accuracy of 79.7%. Taking the mean of scores of the entire polyp section, the classification accuracy increased to 97.3% with an NPV of 95.5% which exceeds the PIVI-1 criteria necessary for a technology to guide the decision to leave in situ colorectal hyperplastic polyps.

The statistical framework in this study focuses on a specific application with limited direct benchmarks, but its performance can still be compared against both OCT-specific and broader AI/imaging applications. Although studies employing OCT imaging and deep learning algorithms have already been demonstrated, with notable success in differentiating benign from invasive or advanced cancerous lesions, these tasks are made considerably easier by the pronounced differences visible in microstructural images. Moreover, the limited research focusing on precancerous lesions has typically involved small patient cohorts and lacked the histological diversity necessary to accurately reflect real-world clinical scenarios. Luo et al. introduced a customized ResNet architecture to classify OCT images as either normal or colorectal cancer, using data from 43 patients. That work reported an AUC of 0.975 but did not incorporate any precancerous lesions [22]. Similarly, Zeng et al. analyzed data from 33 patients to address two classification problems: distinguishing normal from abnormal tissue, with reported sensitivity and specificity of 94.7% and 94.0%, respectively, and differentiating adenomatous polyps from cancerous tissue, achieving 86.9% sensitivity and 85.0% specificity. However, their dataset contained only four adenomatous samples and excluded both hyperplastic tissue and serrated adenomas. Additionally, the study’s use of random train-test splits without ensuring patient-level separation may have led to significant bias, as regions of interest (ROIs) from the same patient could appear in both training and testing sets. This methodological issue introduces the risk of assessment bias by compromising the independence of the validation process [20]. Nie et al. developed a modified ResNet framework to classify OCT images as either malignant potential polyps (adenomas/sessile serrated adenomas) or frank carcinomas, analyzing 35 polyps from 32 patients. However, the study did not include any normal or hyperplastic tissue [23]. Other studies presented promising results using non-OCT AI systems. Zander et al. utilized computer-aided diagnosis systems AI4CRP (82.1% sensitivity, 80.4% accuracy) and compared with CAD EYE software (83.7% accuracy) for diminutive polyp detection [35]. Furthermore, a Narrow Band Imaging (NBI)-based ResNeSt model achieved 95.93% accuracy for hyperplastic polyps with 90.21% sensitivity for deep submucosal cancer [36]. Recently, Okamoto et al., developed a computer-aided diagnosis system using a dataset of 4156 NBI images. Their model was capable of distinguishing between neoplastic and non-neoplastic polyps and could also identify neoplastic polyps with deep submucosal invasion (accuracies from 91.2 to 97.5%) [37]. The findings of the aforementioned studies, using systems other than OCT, suggest that the initial results of our research are highly promising, especially considering that is particularly challenging to distinguish features related to precancerous polyp subtypes.

A limitation constraining the results of this study is the number of participants. The population included in this study should be expanded to include more patients with varying histopathologic diagnoses. Although the 180 patients included in this analysis is more than most studies in the literature, some of the classes, e.g., SSA, were still not well represented. On one hand, this reflects the real-world situation, since polyps were included in the study as they were resected without any prior selection, to avoid bias. However, on the other hand, this imbalance biases the classification algorithms. The problem was partly mitigated with ADASYN data augmentation, but a larger number of patients would still be beneficial. In addition, the images should be annotated by more than one histopathologist to create more precise and verified annotations of the OCT data. Furthermore, this analysis only considers local variations and the statistical distribution of features. Larger scale morphological features must be included in the analysis to provide a more complete representation of the pathology. Finally, given that feature images can be created, their incorporation in deep learning models should also be explored.

Another limitation of this study is the fact that the polyps were imaged *ex vivo*. Both the imaging conditions and the optical properties of the tissue may differ to various extents between the *in vivo* and *ex vivo* environments. However, this study is a necessary first step to establish the feasibility of the procedure. To mitigate the issues pertaining to *ex vivo* imaging as much as possible (i) the tissues were imaged immediately post-excision and kept hydrated with normal saline and (ii) an OCT system compatible with *in vivo* imaging was used. Future work will focus on validating the proposed approach with OCT data acquired *in vivo*, during routine colonoscopy, to assess real-time performance and clinical applicability.

The computational load of the proposed procedure is high, which necessitate the down sampling of the data, to achieve reasonable processing times. However, when the technology is translated to in vivo clinical application, this issue can be mitigated through the use of optimized lightweight algorithms and GPU acceleration. These approaches can significantly reduce inference time, enabling near real-time analysis without compromising diagnostic accuracy. In future work, we aim to integrate such methods to improve the scalability and clinical applicability of our system.

## Conclusions

The use of features extracted from the OCT intensity images, as well as the speckle and spectral content of each neighborhood, shows great promise for the successful classification of pre-malignant changes in colorectal polyps. With accuracies of − 80% per image and − 97% per polyp section and NPVs of 72% and 96%, respectively, the proposed methodology is very promising and warrants further exploration. In the future, the number of patients included will be increased and the algorithms further refined to increase the robustness of the approach. With additional development, this method can offer significant improvements in the accuracy of endoscopic OCT imaging and expand its application in colonoscopic leave-in-situ colon polyp management.

## Materials and methods

### Dataset and pre-processing

**Fig. 4.**
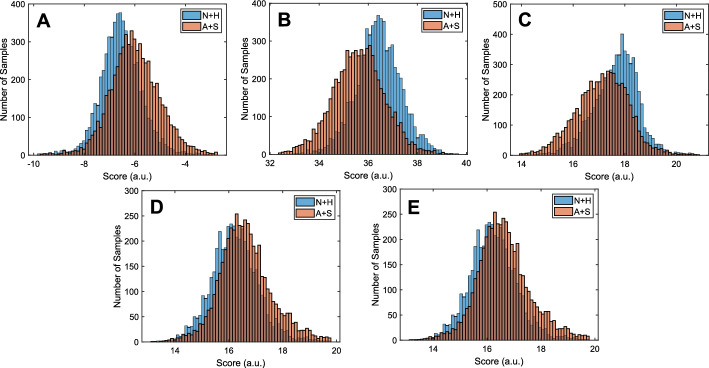
Distribution of calculated scores after ADASYN and MANOVA: (**A**) First order intensity score. (**B**) GLCM score. (**C**) Fractal Score. (**D**) Spectral score. (**E**) GVD score. The horizontal axis is the calculated score and the vertical axis is the number of samples. In all cases the p value was 0 (up to 12 significant digits or more)

**Fig. 5.**
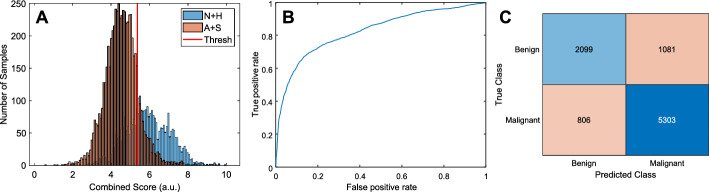
Leave-one-patient-out cross-validation results for benign (i.e., normal and hyperplastic) vs. malignant (i.e., adenoma and SSA) for classification per image (79.69 % accuracy). (**A**) Histogram of the combined score distributions(the horizontal axis is the calculated score and the vertical axis is the number of samples), (**B**) ROC,and (**C**)confusion matrix (180 patients, 9289 images, p value was 0)

The OCT data for this project were collected using a Fourier domain OCT system, with a center wavelength of 1310 nm, an axial resolution of 14 μm, and an A-Scan rate of 100 kHz. The data were collected at the Massachusetts General Hospital after receiving internal review board (IRB) approval. Two hundred volunteers were enrolled in the study. The data from 20 of those patients were removed from the set either due to very poor signal-to-noise ratio (SNR) or severe artifacts due to poor acquisition performance. The final data set included 180 patients with 182 sections, 73 benign, and 109 with malignant potential. Specifically, among the 109 sections with malignant potential, 33 sections were diagnosed as sessile serrated adenomas (SSAs), while the remaining included 76 adenomas. The imaging took place in the colonoscopy suite and the data were acquired immediately after resection of the polyp. Subsequently, a diagnosis was provided from histological examination of H&E sections as per the standard of care. An expert histopathologist used *en face* OCT projections of the polyps to outline the extend of the polyp in the imaging volume as well as annotate areas of histology-confirmed diagnosis (normal, hyperplastic, adenoma, and SSA) to be used as the ground truth (Fig. 4A). Each B-Scan image (Fig. 4B) that was assigned a diagnosis was automatically segmented to retrieve the top portion of the colon mucosa. Segmentation was performed by thresholding, using Otsu’s method, followed by a series of morphological operations to remove small regions due to noise and vertical and horizontal lines due to artifacts. The top surface was identified and the section between the top surface and 150 μm below was segmented (Fig. 4C&D). Both the linear and the logarithmic intensity scale versions of the images were used in the analysis. Both were normalized so that the average intensity value of the top 10% of the pixels was set to 1 (the range selected to avoid the bias of specular reflections), whereas the minimum intensity value was set to 0. Only one of every five images were included in the analysis to (i) assure that all images were completely uncorrelated and (ii) the number of images was reduced to a size that could be processed in a reasonable amount of time with the normal computational power of a personal computer. Given that the classification is performed per image and the imaging step per B-Scan is 5 μm, with a lateral resolution of 25 μm, subsampling does not affect the coverage of continuous lesions. The final data set included 182 polyps and a total of 9289 images. Figure 5 shows some representative image sections of polyps with different histopathologic diagnosis.

### Feature extraction from the OCT data

As already mentioned, much of the information contained in the OCT data remains unseen and underutilized. Fortunately, it is possible to extract various features from the raw and processed data, which can serve as biomarkers of disease [29]. For the purposes of this study, several features were extracted from individual neighborhoods of the image:

- First-order intensity features: First-order intensity statistics, such as the mean, standard deviation, variance, skewness, median, kurtosis, minimum, mode, and maximum of the intensity, were calculated. These features represent the local intensity variations in the image.
- Second-order intensity features: The Gray-Level Co-occurrence Matrix (GLCM) was used. A statistical method for examining texture in images by analyzing the spatial relationship of pixel intensities. It captures how often pairs of pixel values occur at a specified distance and orientation, enabling the extraction of texture features, such as contrast, correlation, energy, and homogeneity. Each GLCM feature was extracted at all directions (0, 45, 90, and 135 °) and for different offsets from the center of the neighborhood (3, 5, and 9 pixels) [30].
- Fractal features: Fractal dimension (FD) was utilized. A quantitative measure of complexity or roughness that describes how the detail in a pattern changes with the scale at which it is measured. Commonly used in image analysis, the FD reflects self-similarity and is useful for characterizing structures with irregular or fragmented shapes, such as biological tissues. The box counting approach was used in this work to calculate the statistics of the FD distribution for each neighborhood in the image [31].
- Scatterer size and spectral scores: A novel metric, the bandwidth of the correlation of the derivative (COD) of the OCT spectrum, has been formulated to estimate the mean size of the scatterers, in this case the nuclei of the cells [26]. It is based on the fact that, according to the Mie theory of light scattering, oscillatory patterns in the spectrum are related to the size of the scatterer. In addition, narrowband intensities of the spectrum and the ratios between those were also calculated for 3, 5, 7, and 15 bands, in analogy to narrowband imaging [12, 13].
- Group velocity dispersion (GVD): A physical phenomenon in wave propagation where different frequency components of a pulse travel at different group velocities, leading to temporal spreading of the pulse. In optics, it is quantified by the second derivative of the refractive index with respect to wavelength, and is critical in ultrafast optics and fiber communication systems. The GVD was measured by examining the speckle patterns in OCT images, a new technique that allows the estimation of GVD in OCT image [24]. Since changes in tissue dispersion are a result of compositional/biochemical alterations, this metric can be an invaluable complement to the microstructural information features.

Each feature was estimated over both an 11 × 11 and an 11 × 22 pixel neighborhood, corresponding to 55 × 55 and 55 × 110 μm, respectively. First- and second-order intensity and fractal features were calculated for both logarithmic and non-logarithmic intensity. The features were calculated only for neighborhoods that had an intensity one standard deviations above the mean to assure that only regions with high nuclear concentrations, therefore higher scattering, were considered during the training process. For each image, the statistics of each feature over the entire image were calculated. This process resulted in eight feature vectors for each image: Log Intensity (180 features), Linear Intensity (180 features), Log GLCM (1200 features), Linear GLCM (1200 features), Log Fractal (180 features), Linear Fractal (180 features), Spectral (3400 features), and GVD (10 features).

**Fig. 6.**
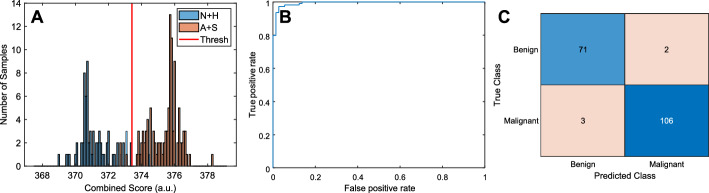
Leave-one-patient-out cross-validation results for benign (i.e., normal and hyperplastic) vs. malignant potential (i.e., adenoma and SSA) for classification per section (97.25 % accuracy).(**A**) Histogram of the combined score distributions per polyp (the horizontal axis is the calculated score and the vertical axis is the number of samples), (**B**) ROC, and (**C**) confusion matrix (180 patients, 182 sections, p value was 0 (up to 65 significant digits))

Some examples of the extracted features are shown in Fig. 6. The feature values are overlaid over the intensity OCT image as a color value in an HSV format, where hue (H) represents the value of the feature (the Hue unit circle representing ± 1σ of the feature values) and value (V) is the intensity of the OCT structural image. The saturation (S) is set to 1 everywhere. These images were created for visualization purposes only. All subsequent analysis was performed on the feature values and their statistics.

### Combination of features to create scores and image classification

Statistical analysis was performed to combine each set of features into a score. Initially, Adaptive Synthetic (ADASYN) sampling was used to create additional samples so that the two classes were balanced [32]. The components of each of the eight feature vectors were ranked according to their predictive power using the Maximum Relevance–Minimum Redundancy (MRMR) algorithm [33]. Subsequently, a subset of features was selected by optimizing the separation of classes using Multivariate Analysis of Variance (MANOVA) [34]. A score was created by a linear combination of the selected features for each neighborhood using the MANOVA-derived eigenvectors. A different score was created for each feature type described above. The process was performed in a leave-one-patient-out (LOPO) manner, i.e., a training set of 179 patients was used to calculate the MANOVA eigenvectors with which the scores of the images of the remaining patient were calculated. This was repeated until scores were calculated for all patients. For each round of the LOPO procedure, the training set score distributions were used to calculate a threshold by estimating the value that maximizes the accuracy of the classification, i.e., the intersection point of the two distributions. The model was simplified by keeping only the features that contributed the top 15% of the predictor score after MRMR. Furthermore, the features that did not contribute to the classification accuracy were iteratively rejected using MANOVA. The features rejected varied at every round of LOPO cross-validation. Overfitting was assessed by comparing the training and the validation accuracies. Given that the average training accuracy was close (~ 5%, depending on the fold) compared to the average validation accuracy of 79.6% the risk of overfitting was considered to be minimal. Using that threshold, the images in the test set were classified as benign (i.e., normal and hyperplastic) or malignant potential (i.e., adenoma and SSA), i.e., above or below the threshold, as illustrated in Fig. 3.

### Combination of features to create scores and polyp section classification

The median of the scores of the images in each polyp was calculated and the classification process was repeated for the entire section. In each case, an overall score was calculated as a combination of the scores calculated above using the following formula:

1 $$S = \sum\limits_{i = 1}^{5} {\sum\limits_{n = 1}^{5} {a_{ni} s_{{_{i} }}^{n} } } + \sum\limits_{i = 1}^{4} {\sum\limits_{j = i + 1}^{5} {\sum\limits_{n = 1}^{5} {b_{ni} \left[ {\frac{{s_{i} }}{{s_{j} }}} \right]^{n} } } } .$$

In each case, *s*_*i*_, is the score corresponding to feature vector *i*. The overall score consisted of a summation of the *n* powers (*n* = *1…5*) of each score as well as the powers of their ratios. As before, the coefficients, *a* and *b*, were calculated using MANOVA to derive the eigenvectors that maximally separate the classes. The process was also performed using LOPO cross-validation where a threshold value was, again, chosen to maximize the accuracy of the classification of the training set. The sections in the training set were, then, classified as benign or malignant potential when below or above the threshold, respectively.

## Supplementary Information

Supplementary Material 1.

## Data Availability

The code and data that support the findings of this article are not publicly available due to privacy and ethical concerns. They can be requested from the corresponding author.
