# Supplementary material for: Classification of colon polyps with malignant potential using statistical analysis of features extracted from ex vivo optical coherence tomography images
Source: Biomed Eng Online. 2025 Sep 26;24:108. doi: 10.1186/s12938-025-01443-z (PMC12465873; doi:10.1186/s12938-025-01443-z)
Supplement: Supplementary file 1 — Supplementary Material 1. [file 12938_2025_1443_MOESM1_ESM.docx]

**Appendix A: Supplemental Material**

**Table S1.** Top 10 most significant features and MANOVA coefficients for each score for the Leave-Patient #26-Out case.

| **Feature / Window Size** | **MANOVA Coefficients** |
| --- | --- |
| **Intensity Statistics – Normalized Logarithmic Images (Total Used: 33 of 180)** | |
| Std(Mean(I)) [21,11] | -1.641 |
| Median(Kurtosis(I)) [11,11] | 1.422 |
| Mean(Skewness(I)) [11,11] | -1.340 |
| Std(Median(I)) [21,11] | 1.190 |
| Mean(Mean(I)) [11,11] | 1.095 |
| Max(Median(I)) [21,11] | -0.741 |
| Max(Skewness(I)) [21,11] | -0.554 |
| Ratio(Max(I)) [21,11] | 0.481 |
| Median(Kurtosis(I)) [21,11] | 0.478 |
| Mean(Kurtosis(I)) [21,11] | -0.465 |
| ⋮ | ⋮ |
| **Intensity Statistics – Normalized Linear Images (Total Used: 22 of 180)** | |
| Std(Median(I)) [21,11] | 2.832 |
| Mode(Median(I)) [21,11] | 2.455 |
| Ratio(Median(I)) [21,11] | 2.417 |
| Max(Median(I)) [21,11] | -2.274 |
| Median(Skewness(I)) [11,11] | -2.129 |
| Max(Min(I)) [11,11] | -0.944 |
| Median(Median(I)) [11,11] | 0.762 |
| Skew(Median(I)) [21,11] | -0.613 |
| Mode(Median(I)) [11,11] | 0.424 |
| Std(Skewness(I)) [11,11] | -0.356 |
| ⋮ | ⋮ |
| **GLCM Feature Saqttistics - Normalized Logarithmic Images (Total Used: 104 of 1200)** | |
| Mean(Correlation-[-3 0]) [11,11] | 29.745 |
| Median(Correlation-[-3 0]) [21,11] | 27.663 |
| Mean(Correlation-[-5 0]) [11,11] | -27.434 |
| Min(Energy-[0 5]) [21,11] | 14.753 |
| Mode(Energy-[-3 3]) [21,11] | -12.260 |
| Median(Correlation-[-5 0]) [21,11] | -11.814 |
| Median(Correlation-[-3 0]) [11,11] | -9.638 |
| Min(AvgHomogeneity-[9]) [21,11] | 8.954 |
| Mode(Energy-[-9 9]) [11,11] | -7.154 |
| Min(Energy-[0 3]) [11,11] | -6.645 |
| ⋮ | ⋮ |
| **GLCM Feature Saqttistics - Normalized Linear Images (Total Used: 79 of 1200)** | |
| Median(Homogeneity-[-3 0]) [21,11] | 16.574 |
| Mean(Homogeneity-[-9 9]) [11,11] | -11.149 |
| Median(Energy-[-9 0]) [21,11] | -6.010 |
| Mean(Correlation-[-9 9]) [11,11] | 4.072 |
| Mode(Homogeneity-[-5 5]) [11,11] | 3.428 |
| Median(Correlation-[-9 0]) [11,11] | 2.556 |
| Mode(Correlation-[-5 -5]) [11,11] | -2.106 |
| Mode(Homogeneity-[-9 -9]) [21,11] | 2.064 |
| Mean(Correlation-[-5 5]) [21,11] | -2.063 |
| Min(Homogeneity-[-9 9]) [11,11] | 1.956 |
| ⋮ | ⋮ |
| **Fractal Dimension Statistics – Normalized Logarithmic Images (Total Used: 25 of 180)** | |
| Var(Mode(FD)) [21,11] | -17.472 |
| Min(Var(FD)) [11,11] | 15.745 |
| Min(Kurtosis(FD)) [11,11] | 7.380 |
| Median(Max(FD)) [21,11] | -5.249 |
| Max(Median(FD)) [21,11] | 3.867 |
| Max(Min(FD)) [11,11] | 3.864 |
| Max(Mode(FD)) [21,11] | 3.642 |
| Mode(Max(FD)) [11,11] | 2.400 |
| Min(Min(FD)) [11,11] | -2.323 |
| Min(Mode(FD)) [11,11] | 2.222 |
| ⋮ | ⋮ |
| **Fractal Dimension Statistics – Normalized Linear Images (Total Used: 67 of 180)** | |
| Min(Min(FD)) [11,11] | 42.996 |
| Mean(Min(FD)) [11,11] | -16.783 |
| Mode(Var(FD)) [11,11] | -11.727 |
| Var(Mode(FD)) [21,11] | 8.969 |
| Mode(Std(FD)) [11,11] | 8.542 |
| Max(Std(FD)) [11,11] | -5.988 |
| Std(Min(FD)) [21,11] | 5.417 |
| Median(Median(FD)) [11,11] | 5.165 |
| Min(Var(FD)) [11,11] | 4.970 |
| Max(Min(FD)) [11,11] | 4.607 |
| ⋮ | ⋮ |
| **Spectral Feature Statistics – Normalized Images (Total Used: 1 of 3400)** | |
| Median(COD) [11,11] | 0.027 |
| **GVD Statistics - Normalized Images (Total Used: 7 of 10)** | |
| Ratio(GVD) [:,11] | 6.521 |
| Kurt(GVD) [:,11] | 1.137 |
| Skew(GVD) [:,11] | 0.290 |
| Mean(GVD) [:,11] | 0.051 |
| Median(GVD) [:,11] | -0.023 |
| Max(GVD) [:,11] | 0.001 |
| Var(GVD) [:,11] | 0.000 |
